# A Derbyshire Practitioner in Answer to Moromastix

**Published:** 1807-10

**Authors:** 


### *To the Editors of the Medical and Physical Journal.*

GENTLEMEN,

FROM a long observance of your impartiality in the admission of papers of controversial Correspondents, I cannot doubt of a similar indulgence being extended to myself; while I hastily run over the observations so indignantly applied to me in your Number of this month.

My object in adverting to a demonstration of the brain, in a paper which I lately submitted to your notice, was not contention; neither did it arise, as suggested by my opponent, out of the fulness of spleen. It is evidently, however, this part of my paper that has so aroused Moromastix, mastix, and which claims therefore a priority of attention from me.

From facts contained in this wrathful ebullition against me, as well as from the indignity of its manner, I cannot but know that it originates at least with the party, whose accuracy I dared (to my great peril seemingly) to call in question. Notwithstanding which, and though I am well able to prove that this dissection, as it respects the brain, was not conducted on the principles of scientific anatomy, yet I shall reserve to myself the privilege of withholding for the present, the particulars of a subject, to disclose which would be but little satisfactory to myself, and would afford, I should fear, still less interest to your various readers; though in a collection like yours, "it surely is a legitimate object of discussion;" and if further urged to it by the indignant contumely of my opponent, I shall not be afraid to offer some facts, which in strictness ought to be appended to this "admirably performed dissection." Nor can I now quit the subject of it altogether, without noticing and doing away some of that misrepresentation which so wilfully abounds in every part of Moromastix's paper. We are told in the first place, "that many medical men of Derby, and its vicinity, attended this admirably performed dissection." Now, from the vicinity, I happened to be the only practitioner that attended; and the town of Derby furnished, besides our operator, two others only, and a resident physician, of character and respectability that would contemn my humble praise. I speak to the day only, on which the brain was the subject of demonstration. With the gentleman who conducted the dissection, I never had any personal intercourse; but I have already acknowledged him of unquestionable respectability, which I felt myself bound to, knowing the opportunities that have been afforded him of medical research. How liberally I am requited. With what profundity, and with what goodness of intention, does Moromastix observe, that, "if a pupil has not attended to his instructions, and profited by them, the fault is not in the Teacher." Greatness of thought! With what propriety I might retort on my opponent this implied censure, is with me no question.

Further, we are led to suppose, that this assemblage of medical men was formed, merely "to see the state of the brain in a person," as he strongly expresses it, "destroyed by hanging." Not, you will observe, to have it generally and scientifically displayed before them, but cooly to to express a desire, simply to see its condition in a person so expiring. Or, in other words, we dispense with an indiscriminating havock of a structure, wonderful in its parts, and admirable in its combinations, to see merely, we suppose he means, its turgent or actually ruptured vessels. Is then Moromastix so antiquated in his opinion on this subject, as to suppose that these miserable sufferers die by the intervention of apoplexy, or that the brain is primarily affected, and is the organ to look to, as accounting in its appearance for all the convulsive struggles and final close of strangulation? "No detailed demonstration," it is continued, "was attempted or given." Now I do contend, without fear of contradiction, that a detailed demonstration was "attempted," though not, I confess, happily "given."

That I have occasionally awkward feelings about me, arising out of conscious professional inferiority, Moromastix, from premises before him, ventures to assert; nor will I controvert him in so happy a position, whose "intellectual eminence," so conspicuous on this occasion, I am obliged feelingly to acknowledge, and whose own reflections, no doubt, arising out of this and other medical rencounters, are all "solacing and triumphant."

By way, I suppose, of dividing our attention, we are told of drawings totally unconnected with the present question; and are reminded of the ingenuity of a gentleman, which no body, knowing his character, could doubt. I am however doubtful, how far ingenuity itself could give us a profitable display of the progressive stages of the operation for aneurism; I hope the knife of the anatomist was not too busy on this occasion; I hope it did not much distort the parts from their "natural position," in preparing them for the pencil.

We dismiss then, for the present, this brainular topic, and proceed to another point of attack, Infantile Diseases.

In my brief mention of infantile diseases, such as were, at that time principally prevailing, it is hardly necessary for me to say, that it was no part of my intention to hold out a fresh or recommendatory view of them to medical practitioners. Surely, Moromastix himself, in the fulness of his animosity, could not so ridiculously depict me in his own mind, as he has attempted to do in his paper, going over, in the compass of a few lines, "the copious subject of infantile diseases." The attempt, as indeed the very face of it will shew, was nothing more than to fix a comparison, as far as my observation enabled me to do it, between the success of medicinal agency, as it regards these little sufferers, in town and in the country; to which I was led by the following emphatic sentence from the pen of a Reporter, then respectfully alluded to. "More infant subjects in this metropolis," says he, "are diurnally destroyed by the mortar and pestle, than in the ancient Bethlehem fell victims in one day to the Herodian massacre." A fact, surely, that merits the consideration of every medical man, and which will be long, I hope, in being appropriately applicable to provincial practice.

The other point, on which Moromastix so impetuously assails me, is, on a case of amputation. The observations which I submitted on this case, are, I hope, in the spirit of candour and liberality, beyond which I set up no pretence for them. By what means does Moromastix endeavour to depreciate them? Not by the force of argument, but by the power of ridicule; the shafts of which, however, are but ill directed towards a subject so seriously important, and so confessedly difficult in its developement.

Our inimitable Disputant, in the ardency of his zeal, takes up the case at a period more early than what I ever adverted to; and talks, but does not shew, the necessity of waiting for the anastomosing branches to dilate before you would proceed to the operation of aneurism. It is not now customary I think, long, if at all, to delay this operation, with the view of allowing the anastomosing branches time to dilate. We cannot, indeed, always determine that such dilatation is going on, even in the advanced periods of the disease; for it is a process we can neither accurately judge of, nor controul; and to protract the operation on that account only, is not acting according to those enlarged views, or that mass of most respectable experience now daily teeming in upon us from the press. Suppose an open wound happening to the femoral artery, Moromastix, this Colossus in surgery, would watch over it, in trembling anxiety, for the collateral arteries to dilate, before he would dare to put a ligature round the main trunk.

It would answer, I fear, but little purpose, closely to trace the steps of Moromastix through all that palpable misrepresentation and abusive epithet, so particularly observable in this part of his paper, and which bury in their exuberance, every argument, if any such originally were. Instead therefore of pursuing so unprofitable a track, let us bring this question to an end by supposing a case.—

Imagine

Imagine a patient laying before you, "in a state of complete deliquium animi;" you perceive, without previous question, that this death-like appearance originates in hæmorrhage; you hastily make yourself acquainted with the former history of the patient; you learn the source of this bleeding, and are told also, "that after the most severe pressure which possibly could be made, (by the tourniquet) he lost some blood;" you conclude, therefore, that its present cessation is more owing to the quiescence of the heart and arteries than to the pressure of the tourniquet, which we will suppose still grasping the limb; you know, from previous circumstances, that this hæmorrhage could not be in a direct line from the main trunk,—an important fact; and you know also, that it filters through a diseased or sloughy surface, through "a mass of corruption," in which condition of parts it would be in vain to look out for the bleeding orifice; and as the patient revives, you apprehend a return of hæmorrhage, which, in ever so small a degree, you have reason to think would prove fatal. Such a case, it must be allowed, would require promptness of decision, and would be variously encountered perhaps by different surgeons; in the headmost rank of which, if not for ingenuity of practice, at least for novelty of opinion, stands Moromastix, who, it seems, would have "amputated the limb, as the only chance of saving it." Rather than have amputated a thigh in such a precarious state of the patient, I hope there is nothing radically wrong in having suggested, "That we would have dilated the cyst if it required it, and would have crammed it with compresses of sponge; and with the help of a bandage, or other means, would have kept up a necessary resistance to the bleeding point or surface." If success could not be promised from such practice, does it become on that account only, less rational? And would it not be advisable to hazard every reasonable expedient, rather than to hurry, by the horrors of mutilation, a destined sufferer to his grave.

This kind of procedure, "as applicable to such a case," is called by Moromastix, somewhat in the spirit of tautology, "preposterous nonsense." And again, "what bombast." Epithets of this sort, we would remind him, are easy of selection, and may be applied by almost any ignoramus, in the way of ridicule or degradation, to the most serious and important topics. It must be something, however, a little more argumentative that brings conviction to my mind, or that makes me to think that "a state

 of of complete deliquium animi," from whatever cause arising, is the moment for the introduction of the parade of amputation; or rather, I should say, the moment for actual operation.

A PRACTITIONER IN DERBYSHIRE.

*August* 26, 1807.

